# A wrong conclusion

**DOI:** 10.1186/s13049-024-01200-6

**Published:** 2024-04-09

**Authors:** Hadi Mirfazaelian

**Affiliations:** 1 Emergency Medicine Department, Tehran University of Medical Sciences, Tehran, Iran; 2 Prehospital and Hospital Emergency Research Center, Tehran, Iran

Dear Editor,

I read the study published by Nikula et al. [1] with interest. As provided by the authors, “the objective of this study was to evaluate whether intranasal dexmedetomidine could provide more effective analgesia and sedation during a painful procedure than intranasal ketamine” [1]. As depicted in the statistical analysis section, it is a superiority trial and hence the null (H0) hypothesis should be “dexmedetomidine is not superior to esketaime”. By conducting this study, the investigators tried to reject the null hypothesis and conclude that it is superior to ketamine (H1 hypothesis).

In the conclusion, they stated that “This study was underpowered and did not show any difference between intranasal dexmedetomidine and intranasal esketamine for procedural sedation and analgesia in young children.“ [1]. I have 2 arguments; first, although early stoppage of a trial would generally reduce the power [2, 3], this can be stated only after post-hoc power analysis. Second, the inability to reject the null hypothesis should lead to a conclusion that the study failed to demonstrate the superiority of dexmedetomidine over esketamine. As a result, it is more accurate for conclusion to be read as “the results failed to show that Dexmedetomidine was superior to the esketamine” or “reduction in pain as per FLACC, was not statistically significant”. In my view, the conclusions drawn by the authors do not accurately reflect the statistical findings, potentially leading to misinterpretation of the study’s implications.

Sincerely yours,

Hadi Mirfazaelian MSc, MD.
